# 429. Infection Prevention During Use of a Warm Zone Model in Cohort Patient Care Locations during the COVID-19 Pandemic

**DOI:** 10.1093/ofid/ofab466.629

**Published:** 2021-12-04

**Authors:** Joseph Sacht, Samantha Holton, Mylinh Yun, Jay Varkey

**Affiliations:** 1 Emory University Hospital, Atlanta, Georgia; 2 Emory Healthcare, ATLANTA, Georgia

## Abstract

**Background:**

The COVID-19 pandemic required hospitals to care for influxes of patients in cohort locations during critical shortages of personal protective equipment (PPE). Safety zones can be used to protect healthcare workers caring for patients with infectious pathogens. During the COVID-19 pandemic, our hospital developed a Warm Zone model (WZM) to streamline the care of patients with COVID. We established specific areas in our COVID cohort units where staff were permitted to bridge between rooms without doffing gowns, but still doffing gloves and performing hand hygiene between patients. We recognized that a WZM could inadvertently increase risk of nosocomial transmission of pathogens if gowns acted as fomites. For this reason, patients with known infectious pathogens were excluded from the WZM. To measure for unintended harmful consequences of the WZM, our Infection Prevention (IP) department performed surveillance for hospital onset (HO) Clostridioides difficile (CDI), Carbapenem-resistant enterobacteriaceae (CRE) and Methicillin-resistant Staphyloccocus aureus (MRSA) bloodstream infections on units that implemented the WZM.

**Methods:**

Two intensive care units and 3 wards where COVID positive patients were cohorted were included in surveillance. The timeframe for this analysis was 7/1/2020 - 3/31/2021. An electronic surveillance system was used to measure HO infections. The National Healthcare Surveillance Network (NHSN) LabID definitions were used when determining HO CDI and MRSA bloodstream infections (BSI).

**Results:**

During the study period, there were no HO CRE, 1 HO CDI, and 2 HO MRSA BSI in cohort units. There was no evidence to suggest that the HO CDI or HO MRSA BSI were associated with use of a WZM. During this time period, there were 14 cases of community onset (CO) CDI, 2 cases of CO MRSA BSI, and one CO CRE.

**Conclusion:**

During use of a WZM in COVID cohort units, IP did not identify significant increase in HO CDI, CRE, or MRSA BSI compared to non-cohort units. We were limited in our ability to measure acquisition of pathogens because active surveillance screening for colonization was not performed. However, we were able to safely employ a WZM to streamline patient care in COVID cohort areas without evidence of causing nosocomial infections via patient-to-patient transmission.

**Disclosures:**

**All Authors**: No reported disclosures

